# Defining continuity of care from the perspectives of mental health service users and professionals: an exploratory, comparative study

**DOI:** 10.1111/hex.12435

**Published:** 2015-12-29

**Authors:** Angela Sweeney, Jonathon Davies, Susan McLaren, Margaret Whittock, Ferew Lemma, Ruth Belling, Sarah Clement, Tom Burns, Jocelyn Catty, Ian Rees Jones, Diana Rose, Til Wykes

**Affiliations:** ^1^ Population Health InstituteSt George's University of LondonLondonUK; ^2^ Department of ChildFamily and Community StudiesDouglas CollegeBritish ColumbiaCanada; ^3^ Faculty of Health and Social CareLondon South Bank UniversityLondonUK; ^4^ Department of Health Service and Population ResearchInstitute of PsychiatryPsychology and NeuroscienceKing's College LondonLondonUK; ^5^ Department of PsychiatryUniversity of OxfordOxfordUK; ^6^ Tavistock CentreLondonUK; ^7^ WISERDSchool of Social SciencesCardiff UniversityCardiffUK; ^8^ Department of PsychologyPsychology and NeuroscienceInstitute of PsychiatryKing's College LondonLondonUK

**Keywords:** continuity of care, general adult psychiatry, health professionals, mental health services, service users

## Abstract

**Background:**

Continuity of care (COC) is central to the organization and delivery of mental health services. Traditional definitions have excluded service users, and this lack of involvement has been linked to poor conceptual clarity surrounding the term. Consequently, very little is known about the differences and similarities in the conceptualization of COC by mental health service users and professionals.

**Objective:**

To explore and compare mental health service users’ and professionals’ definitions of COC.

**Methods:**

Using an exploratory, qualitative design, five focus groups with 32 service users each met twice. Data were analysed thematically to generate a service user‐defined model of COC. In a cross‐sectional survey, health and social care professionals (*n* = 184) defined COC; responses were analysed thematically. Service user and professional definitions were conceptually mapped and compared to identify similarities and differences.

**Results:**

There was crossover between the service user and professional derived models of COC. Both contained temporal, quality, systemic, staff, hospital and needs‐related elements of COC. Service users prioritized access, information, peer support and avoiding services; health professionals most frequently referred to staff, cross‐sectional and temporal COC. Service users alone identified service avoidance, peer support and day centres as COC elements; professionals alone identified cross‐sectional working.

**Conclusions:**

Important similarities and differences exist in service user and professional conceptualizations of COC. Further research is necessary to explore these differences, prior to integrating service user and professional perspectives in a validated COC framework which could enable the development and evaluation of interventions to improve COC, informing policy and practice.

## Background

In response to deinstitutionalization and an expanding body of international evidence on service fragmentation, achieving continuity of care (COC) has become a key challenge facing mental health services.^1^ Although COC has a shared intuitive meaning, with overarching definitions emphasizing cohesion, smoothness and connectedness,^2^ agreement regarding its specific definition is lacking.^3^ Consequently, COC has been described as ‘a conceptually underdeveloped, vague and overinclusive construct lacking a solid empirical foundation’.^3^ As a result, COC is infrequently defined in exploratory and applied studies^4^ making it problematic to develop, measure and compare interventions to improve it.

Poor clarity in the conceptualization and operationalization of COC has been linked to a lack of service user involvement.^5^ Historically, COC definitions have been dominated by the perspectives of professionals, and it has typically been assumed that service users and professionals define COC in the same way.^4^ This has recently been described as the ‘Professional Paradigm’.^6^ However, there is evidence that service users and professionals understand and prioritize service elements differently.^7^ For example, a qualitative metasynthesis of studies of patients’ perspectives conducted in a range of service settings found that service users’ emphasized communication, information transfer, accessibility and relational COC with a single health professional.^8^ Of the twenty‐five papers selected for inclusion in this metasynthesis, only three had investigated mental health service users perspectives on COC, illustrating the relative paucity of information relating to this group. In contrast, it has been suggested that health professionals view COC as information sharing and a personal relationship with the service user, influenced by wider policy and resource issues.^9^ In a ‘Perspectivist Paradigm’, service users’ views and experiences are valued, with professionals views elicited for the extent to which they correspond with service users’ perspectives, and for their insights into organizational issues.^6^ More recently, a ‘Partnership Paradigm’ has been proposed whereby ‘care is co‐constructed through the interaction between patients, members of their informal care networks and professionals’, suggesting that mental health COC research can move towards the exploration and understanding of the co‐production of COC as it is enacted through relationships.^6^ This should be underpinned by the conceptualizations of both service users and professionals.

Writing in the field of chronic disease, Naithani and colleagues have drawn a distinction between continuity in the delivery of care, which encompasses COC elements that are relevant to care providers, and continuity in the experience of care, which concerns the knowledge and priorities of service users and their families.^10^ This is reflected in the Freeman model of COC which prioritizes e*xperienced COC,* meaning that service users experience the progression of care as smooth and co‐ordinated, enabled by the following elements of continuity of delivery: *relational; longitudinal; flexible; cross‐boundary/team;* and *informational*.^4^ A subsequent adaptation to mental health added *long‐term* and *contextual* COC (enabling people to sustain social relationships and quality of life).^11^

Despite service users’ experiences of and satisfaction with mental health services increasingly being placed at the heart of service development and provision,^12^ little is known about how mental health service users conceptualize experienced COC. Moreover, given the paucity of comparative studies, a need exists to determine how mental health service user and professional definitions of COC either converge or differ so that services are not organized and delivered according to the Professional Paradigm alone. Consequently, this study aimed to explore and compare service user and professional definitions of COC. The study was conducted as part of a wider programme of research which aimed to investigate experiences of COC and relationships to health and social outcomes (The ECHO study: Experiences of Continuity of Care and Health and Social Outcomes).^13^, ^14^ Within this broad research programme, participatory research with service users explored their definitions, perspectives and experiences of COC with the primary aim of generating an outcome measure of experienced COC (CONTINU‐UM).^15^, ^16^ The views of professionals were explored using survey methods in a separate strand in order to investigate organizational factors influencing COC.^17^ The current study brought these two strands of work together through conceptual mapping and narrative analysis of professional and service user‐defined models of COC.

## Methods

### Setting

An exploratory qualitative design was implemented in Community Mental Health Teams (CMHTs) in two National Health Service (NHS) Mental Health Trusts in London within the timescale of a broader programme of COC research (The ECHO Study, 2001–2007).^5^, ^13^, ^14^, ^15^, ^16^, ^17^ The Trusts were based in two inner‐city areas with high Jarman indices and a suburban area with a lower Jarman index in order to recruit service users from different sociodemographic groups.

Within UK adult mental health services, health and social care are integrated and COC is an important quality benchmark. CMHTs incorporate the skills of a range of health and social care professionals to deliver and coordinate diverse services through integrated working in generic and specialist teams. Initiated to address concerns over service fragmentation, poor interdisciplinary communication, decision making and negative service user experiences,^18^, ^19^, ^20^, ^21^ the vision has been to provide a seamless service characterized by improved access, removal of gaps and effective care co‐ordination.^22^ Although implementation of integrated working has been marked by challenges and benefits,^23^ fewer deaths, lower levels of service user dissatisfaction with care and fewer hospital admissions have been reported.^24^

### Ethical approval

Full ethics approvals were granted by South London and Maudsley/Institute of Psychiatry Ethics Committee (reference 128/01) and Wandsworth Research Ethics Committee (reference 01.42.8).

### Service user participants

Given that the overarching aim of the ECHO study was to investigate experiences of COC and health and social outcomes, focus and clarity regarding outcomes was vital. A decision was made to focus on service user participants with a diagnosis of psychosis in order to reduce the heterogeneity of treatment patterns and reduce ‘noise’ so that any patterns could be clearly identified. Participants diagnosed with psychosis were also chosen because it was assumed that they would have complex needs and therefore have experiences of cross‐sectional COC (i.e. of moving within and between services). An extension to the ECHO study investigated the COC experiences of service users’ who did not have diagnoses of psychosis.^25^

Participants were recruited from local CMHTs, service user groups and day centres. All CMHT service users eligible for inclusion were invited to participate via information sheets distributed by CMHT staff. Those considering participation contacted researchers directly on an autonomous voluntary basis. Service user groups and day centres were visited by (*author initials)* and those interested were able to discuss the nature of participation. Inclusion criteria were: (i) diagnosis of psychosis (ii) aged 18–65; and (iii) in contact with services for at least 2 years. The latter criterion ensured that participants with experiences of longitudinal COC (i.e. care over time) were included.

### Professional participants

Participants in managerial and frontline operational roles were recruited from 19 CMHTs and associated acute units within the NHS Mental Health Trusts. One CMHT declined to take part due to workforce pressures. Both Trusts had integrated health and social care delivery by CMHTs and had implemented the Care Programme Approach. The sampling framework for the survey component comprised the total population (*n* = 276) of health and social care professionals responsible for the delivery of COC: those on long‐term leave or training were excluded. Overall, the survey response rate was 70% (*n* = 192/276) and the item response rate for the question asking respondents to define COC was 94% (*n* = 184/192).

### Service user data collection

Five focus groups were each held on two occasions (initial and repeat) with a total of 32 service users participating. Written informed consent was given prior to participation. Groups had between 4 and 12 participants, were facilitated by two service user researchers (*authors’ initials)* and lasted approximately 2 h. All groups were held in settings that were comfortable and familiar to participants. Initial groups opened with participants telling their stories of their first contacts with mental health services. Participants then discussed experiences of services and definitions of COC based on a topic guide which included relationships with key staff members (e.g. what did and did not work well, continuity of contact), support services and how these fit together, support needs in a crisis and gaps in care. Groups were audio‐recorded, transcribed by an independent transcriber and analysed thematically.^26^ Repeat groups began with member checking through a detailed discussion of the interim thematic analysis.^27^ Participants then ranked COC elements (extracted from the thematic analysis of the initial group) individually and collectively and data were again analysed thematically as above. Finally, the thematic analysis, service users’ explicit definitions of COC and individual and group ranking results were compared to generate a service user‐defined model of COC. Through a series of Expert Panels (*n* = 12) and consultations (*n* = 3), the model was developed into an outcome measure (CONTINU‐UM) and validated in a field trial (*n* = 167).^15^

### Professional data collection

Data were collected utilizing a postal questionnaire designed *de novo*; this comprised ten sections in which a mix of Likert scaled and open ended questions investigated definitions and experiences of COC. One open ended question asked respondents to define COC in their own words. The questionnaire met requirements for content validity, test–retest reliability (Spearman Brown coefficients: range 0.64–0.96) and internal consistency (Cronbach's alpha coefficient: 0.92) and took 12–18 min to complete. Questionnaires were distributed at CMHT meetings for self‐completion and return via a drop box.^13^ Data was extracted and entered on a computer spreadsheet and the extraction process independently checked for error prior to analysis.

### Data analysis

#### Stage 1: thematic analysis of service user data

In the first stage of analysis, focus group transcripts were repeatedly read by (*author's initials)* for familiarization and to develop an initial coding frame. The coding frame focussed on potential continuity definitions, practical suggestions to improve continuity, points of heavy debate or consensus, patterns within and across transcripts, and early interpretations and ideas. The coding frame was applied to the data using MAXqda software by (*author's initials*), with all codes following Boyatzis definition of a ‘good code’.^28^ Two focus groups were additionally analysed by (*author's initials)* to deepen insight into the data through discussion and comparison of themes, a form of multiple coding.^29^ Through a cyclical process of coding new data, reading both the content of themes and the entire data set, codes were redefined, combined and expanded.^28^ Consequently, transcripts were coded and recoded until firstly, the coding frame appeared to account for what was occurring in the data, and secondly, the codes were internally consistent, discrete and being applied consistently. This process continued until a coherent coding frame was developed that appeared to capture what was occurring across the data set and that related to the aims and objectives of the analysis, resulting in a final list of COC themes.

#### Stage 2: thematic analysis of professional data

In the second stage of the analysis, responses to the key question were read by (*author's initials)* to generate initial thematic COC definitions.^26^ Themes were developed and refined by a small, multidisciplinary research team (*authors’ initials x3*). A revised thematic framework was applied to the data by (*author's initials)* though a cyclical process of reading survey responses and generating, applying, expanding and redefining COC labels.^28^ To enhance validity, two researchers (*author's initials)* independently double coded one‐third of the data set.^27^ Revisions were discussed collaboratively leading to a final thematic framework which was reapplied to the dataset.

#### Stage 3: conceptual mapping and narrative comparison of service user and professional defined COC models

In the final stage of the analysis, a modified form of conceptual mapping was used to map and interpret interrelationships amongst key COC concepts that had emerged from the thematic analyses in stages 1 and 2.^30^, ^31^ Conceptual mapping was employed because it enables exploration of interconnections and differences across large amounts of data and multiple studies.^31^ A map of each model was created containing each element of COC, its meaning and the content derived through thematic analysis. A tabulated grid was then generated which contained primary data, initial ideas, final coding, conceptual mapping results and further conceptual mapping against two pre‐existing models of COC.^11^, ^32^, ^33^ This enabled us to view relevant, condensed data from multiple cases in a single format for further exploratory analysis, alongside wider literature. We then systematically compared the service user and professional models of COC in order to gain an understanding of their similarities and differences. The models were compared and contrasted in an iterative process, interrogating their meaning, identifying and expounding connections and points of divergence, understanding the location of the emerging findings in the wider literature. To enhance validity, emerging results were discussed by the research team which led to further understanding and explication of the similarities and differences between the service user and professional models of COC.

## Results

### Service users

Focus group participants’ (*n* = 32) mean age was 47 years; 40% were female, 24% were from a minority ethnic background and the mean length of contact with services was 16 years. The final service user‐defined model of COC contained 16 elements: easy *access* to services; *range* of needed services; *waiting* for services; *out of hours* support; support from services following *hospital discharge*; infrequent *staff changes*; appropriate *information* from staff; service *flexibility*; services enable *individual progress*; suitable *day centres*; agreed *care plan*;* crisis systems*;* communication between staff*;* peer support*; not having to *repeat your life history*; and *avoiding contact with services*. (For further information on definitions see Table 1).

**Table 1:** Health professionals definitions of continuity of care by occupational group^a^ (Acronyms: see footnote)

Definition of Continuity	CPN *n* = 51 *n* (%)	SW *n* = 44 *n* (%)	PSYCH *n* = 33 *n* (%)	RMN *n* = 25 *n* (%)	PSY *n* = 14 *n* (%)	OT *n* = 10 *n* (%)	HCA *n* = 6 *n* (%)	Total *n* = 184 *n* (%)^b^
Staff: continuity between staff/teams	11 (22)	13 (30)	19 (58)	14 (56)	8 (57)	1 (10)	3 (67)	70 (38)
Temporal: meeting care needs over time	16 (31)	14 (32)	14 (42)	4 (16)	3 (21)	0 (0)	1 (17)	54 (29)
Cross‐sectional: multi‐agency care	14 (27)	13 (30)	5 (15)	7 (28)	9 (64)	4 (40)	1 (17)	53 (29)
Broad definitions: consistent, cohesive or seamless care	16 (31)	12 (27)	8 (24)	3 (12)	3 (21)	5 (50)	0 (0)	47 (26)
Process of delivery: procedures enabling continuity of care	20 (39)	6 (14)	4 (12)	4 (16)	3 (21)	2 (20)	0 (0)	39 (21)
Hospital admission/discharge: continuity across inpatient admissions and discharges	9 (18)	9 (20)	4 (12)	10 (40)	2 (14)	2 (20)	2 (33)	38 (21)
Meeting needs: care meeting individual and community needs	12 (23)	6 (14)	7 (21)	3 (12)	3 (21)	3 (30)	0 (0)	34 (18)
Quality: evaluated, effective care	5 (10)	7 (16)	4 (12)	1 (4)	2 (14)	3 (30)	2 (33)	24 (13)
Negative definitions: absence of breaks or gaps in care	9 (18)	6 (14)	2 (6)	1 (4)	2 (14)	0 (0)	0 (0)	20 (11)
Service user, carer and wider networks: involvement and inclusivity	3 (6)	10 (23)	5 (15)	2 (8)	1 (4)	0 (0)	0 (0)	21 (11)
Information: information sharing between key groups	2 (4)	2 (5)	3 (9)	1 (4)	1 (4)	1 (10)	0 (0)	10 (5)
Access: rapid, easy access to care	0 (0)	2 (5)	0 (0)	0 (0)	0 (0)	0 (0)	0 (0)	2 (1)

a Acronyms: CPN (Community Psychiatric Nurse); SW (Social Worker); PSYCH (Psychiatrist); PSY (Psychologist); RMN (Registered Mental Nurse); OT (Occupational Therapist); HCA (Health‐care Assistant).

b Frequencies expressed as a % of the total population (*n* = 184).

### Professionals

The item response rate for the question asking respondents to define COC was 94% (*n* = 184/192). Of these respondents, fifty‐seven per cent were female; 42% were from minority ethnic backgrounds and time in current post ranged from one to 15 years. Occupational groupings were dominated by nurses and social workers.

Twelve elements of COC were identified: *staff*;* cross‐sectional*;* temporal*;* broad definitions; process of delivery; hospital admission and discharge; meeting needs; quality; negative definitions; service user, carer and wider networks; information;* and *access*. (For further information on definitions see Table 1). The most frequently cited elements were staff, cross‐sectional and temporal COC while those least frequently cited were continuity of information and access. Broad definitions of COC described it as consistent, smooth, cohesive or seamless, in contrast to negative definitions identifying the absence of breaks or gaps in care. Few definitions referred to service quality.

Within occupational groups, the most frequently cited elements were: staff and cross‐sectional COC (psychologists); staff and temporal COC (psychiatrists); and staff, hospital admission and discharge (psychiatric nurses). Social workers cited service user, carer and wider networks as a definition of COC more than any other group, while processes of care delivery were most commonly cited by CPNs. Access was cited solely by social workers; occupational therapists rarely mentioned staff and temporal COC; psychiatrists infrequently mentioned cross‐sectional COC and few psychiatric nurses identified temporal elements.

### Conceptual mapping and narrative comparison of service user and professional defined COC models

Several elements of COC had immediate cross‐model equivalents: these were meeting needs; mechanisms of care delivery; staff; hospitalization; information; access to services; temporal aspects; and service quality (see Table 2). Similar elements of COC were at times conceptualized differently by each group. For example, regarding meeting needs, service users considered individual needs, *‘services help people to progress on their terms’;* while staff additionally considered local population needs, *‘providing an effective service to meet the identified needs of the patient population’*. When conceptualizing wider networks, professionals emphasized user and carer involvement in care delivery, *‘care across services as experienced by user…. on‐going services to meet needs of service users and carers’;* while service users emphasized peer support ‘*I've experienced more support from users than from professionals.’* There were also key differences between models. Most notably, informational COC and access to services were important to service users but were infrequently identified by professionals. Day centres and avoiding services only appeared in the service user model and cross‐sectional COC only in the professional model.

**Table 2:** Comparison and crossover: service user and health professional definitions of continuity of care (COC)

Health professional exemplar definitions	Health professional codes	Health professional defined elements of COC	Service user‐defined elements of COC	Service user codes	Service user exemplar definitions
*‘Ensuring that all health/social care agencies work together in co‐ordinated fashion in providing care to clients.’*	Transitions, integration, coordination, multi‐agency working, working across health and social care.	Cross‐sectional	–		–
–	–	–	Day centres	Providing daily activities, social contact and support.	*‘Going to day centres is good because it means I've got somewhere to go during the daytime, instead of just kicking around the house all day and feeling down.’*
–	–	–	Avoiding services	Service users control of COC, ability to have *dis*continuity.	*‘Well, if you break away from that treatment, you're subject to, possibly, being forcibly having your liberty taken away from you.’*
*‘Represents the optimum of patient care both in a spatial and temporal sense where there is a continuance of content and input and exchange between patient, carers, and professionals.’*	Care until discharge, on‐going care, aftercare	Temporal	Waiting Out of hours	Not having to wait for needed services, particularly in a crisis. Being able to access support from services 24/7, preferably from a known person.	*‘Knowing I haven't got the problem of waiting for the psychiatrist.’*
*‘Providing high‐quality, consistent level of care with adequate resources to meet objectives of an individual's care plan.’*	Effective, professional, appropriate, safe, proper, evaluated	Quality	Implicit to the service user model		
*‘Providing an effective service to meet the identified needs of the patient population and ensuring that an individual receives care based on assessed needs rather than service availability, etc.’*	Population needs, holistic care, health promotion, flexibility, quality of life, providing the range of services needed	Meeting needs	Flexibility Individual progress Range	Services responding swiftly to changing needs. Services help people to progress on their terms. Not having gaps in care, having all the services you need.	*‘Continuity of care means that you progress yourself … acting positively, and thinking positively, and making the person who is ill a part of society again.’*
*‘Better liaison with other disciplines and agencies. Proper assessment and implementation and evaluation of care’*	Assessments, treatment plans, care plans, packages of care, care pathways	Process of delivery	Care plans Crisis systems	Flexible, agreed with service user, on discharge from hospital. Lack of support from services, important role of family and friends, need for crisis systems, need to talk to a professional who will listen.	*‘It needs to be managed with the help of a properly constructed care plan – for you, not for anyone else, for you, individually.’*
*‘To provide a service where clients do not have to change workers too often and do not feel left without help.’*	Staff changes, handovers, communication between staff, liaison, team working	Staff	Staff communication Staff changes: Repeating your life history	Importance of staff communication, service users to know what is said. Frequent staff changes, handovers, staff changes sometimes welcomed. Repeating life history to new staff members.	*‘You don't want staff to change, and when they do, you don't want a black hole left there. You want stability.’* *‘I get fed up with giving my history to everybody.’*
*‘Care across services as experienced by user. Preventative work – on‐going services to meet needs of service users and carers to include CPA.’*	Service user involvement, carer involvement, social inclusivity	Service user, carer and wider networks	Peer support	Importance of peer support.	*‘I've experienced more support from users than from professionals.’*
*‘Seamless care of patient from inpatient to outpatient, across services, primary and secondary – across professions. Health and social services etc.’*	Care following through sites, follow‐up support	Hospital admission and discharge	Hospital discharge	Support on discharge from hospital, discharge planning.	*‘I was in bed they turned up and say, ‘Eh, you have to get up, there's a cab waiting for you to take you to the housing’, and that's it – that's how they discharged me.’*
*‘Seamless transition of patient information, care passed from members of staff to another, or team to another with little or no disruption to the package of care offered to the patient.’*	Between professionals, to service users	Information	Information Staff communication	Receiving the information you need from staff, information to help close gaps in care. Don't know whether staff communicate or what about, poor communication causing breaks in care.	*‘If for example you don't know that the clinic exists, you can't ever go and ask your psychiatrist for it, but as soon as you do ask him, he'll say “Yeah, ok, I'll put you on the list.”’*
*‘Care for patients in the community with a) support and b) same day structure’*	To services	Access	Access	To services, particularly in a crisis, staff as gatekeepers, not being believed.	*‘Well, services don't come to you, not just like that! You've got to search them out!’*

## Discussion

This study is the first to explicitly compare the definitions of COC generated by mental health service users and professionals. We found some convergence between service user and professional models of COC, with both groups seeing COC as functioning to meet people's needs by providing the necessary range of services with flexibility and the aim of helping people to progress in their lives, or maintain a good quality of life.

However, there were also key differences and tensions between the models. Four elements of COC were identified by service users but rarely or never by professionals. First, service users identified easy access to services as crucial to a needs‐responsive service; in contrast, access was only cited by two professionals. While including access as an element of COC is sometimes considered contentious,^34^ it features in the majority of multidimensional COC definitions^3^, ^32^, ^35^, ^36^ and Reith has observed, ‘Although there is often talk of a seamless service, what this must be made to mean in reality is not using boundaries to restrict a person's access to a service he or she requires’.^37^ It could be argued that access should be removed from a conceptualization of COC because it *facilitates* COC, rather than defining it. However, the same can be said of staff communication, yet this is never excluded from existing definitions. To service users, it is ease of access that predominantly determines COC experiences, making it fundamental to service user‐defined COC.

Second, service users emphasized the importance of support from others who had experienced mental distress as an important aspect of COC – often over and above support from staff, friends and family – yet this was not identified by professionals, despite the growing significance of peer support worker roles.^38^, ^39^

Third, service user participants argued very strongly that day centres should be included in a definition of COC because experiences of isolation had severely exacerbated their mental health problems. This meant that for many, day centres met an important need for social contact; this is defined by Freeman as contextual COC, or continuity of social context.^11^ That professionals did not identify day centres may be because they underestimated the need for daytime and out of hours COC or the role of day centres in filling this need, because they adopted a narrower definition of COC focussed on statutory services, such as CMHTs and inpatient wards, or because their views reflect the significant decline of day centres in England.^40^

Finally, the service user‐defined model included service avoidance: in negative avoidance, service users avoided services because they did not realize they needed support, or because they feared the loss of choice and control. In positive avoidance, service users had developed their own strategies for living and no longer wanted or needed services. This contrasts with the mental health COC literature which sees discontinuity almost exclusively as dangerous or harmful,^37^ and from which day centres, peer support and avoiding services are almost entirely absent.^3^, ^4^, ^32^, ^35^ Inclusion of service avoidance in COC models has been controversial, since some expert views are that studies cannot evaluate interventions aimed at improving COC where outcomes assess both COC and discontinuity of care. It can be argued that avoiding services is integral to service user‐defined COC as supported by the ‘Partnership Paradigm’.^6^ As a minimum, service users ability to control COC and have discontinuity of service contacts should be included in any protocols encompassing the implementation of guidelines, interventions or activities to enhance COC.

In contrast, while professionals frequently referred to cross‐sectional COC, emphasizing procedures and processes, this element was not identified by service users. This may in part be understood in the context of the survey timing which was conducted shortly after the local integration of health and social care services, underpinned by policy directives on collaborative working.^40^ Professionals also stressed the importance of staff COC, reflecting contemporary policy drivers,^40^ while service users sometimes welcomed staff changes where relationships were difficult. Within professional groups, the predominance of staff COC identified by more than half the psychiatrists and psychiatric nurses could reflect closer working contact with service users. Similarly, many social workers identified the importance of cross‐sectional, multiagency care together with wider user and carer networks, a reflection of professional expertise.

Overall, our findings suggest that current conceptualizations of COC do not adequately account for the range and emphasis of definitions highlighted by either mental health service users or professionals. However, most notably, the extant mental health COC literature rarely addresses the concept of positive service avoidance, nor does it acknowledge the importance and relevance of peer support and day centres. There is some overlap between the service user and professional models of COC and prior definitions. For instance, early COC operationalizations occurred in the context of deinstitutionalization and focussed almost exclusively on hospital admission and discharge;^34^ this clearly remains important to service users and professionals. Relational, cross‐sectional and informational COC, and accessibility and flexibility can also be found in other COC models.^2^, ^3^, ^4^, ^11^, ^32^, ^35^, ^36^ Findings are consistent with an earlier view that definitions and experiences of continuity differ between service users and professionals and there is some crossover with the multidisciplinary definition of COC encompassing informational, managerial and relational dimensions, although service users and professionals emphasized elements of these components differently.^41^ However, including the views of service users often means understanding these dimensions from a new perspective. For instance, relational COC (present in the professional model as well as broader models) was expanded by service users to include peer support. Informational continuity was expanded by both service users and professionals from ‘information follows service users’^11^ to encompass service users own access to information and/or the provision of information to service users by staff. Thus, service users conceptualize some continuity elements similarly to professionals and pre‐existing models, reconceptualize some elements from the perspective of receiving rather than providing services and also identify aspects of continuity that are unique to service users. Yet despite this, it remains rare for service users’ perspectives to be included in models and operationalizations of COC, and where service users’ views are elicited it is often difficult to separate them from those of professionals.^3^, ^4^, ^11^, ^35^, ^36^

### Continuity of care or good quality care?

Freeman and colleagues have noted that continuity is both broad and fluid, making it difficult to generate a firm definition. Like other social constructs, COC can be considered a ‘fuzzy concept’ which means that finding hard conceptual boundaries can be problematic.^42^ Despite this conceptual uncertainty, researchers have not yet explored the conceptual boundaries around COC.^1^ Clarifying conceptual boundaries has therefore been described as one of three key challenges for COC researchers.^43^

Perhaps the haziest boundary is that between COC and quality of care. Continuity and quality are entangled in many authors’ work. For example, Bachrach believes that services striving to achieve COC are characterized by excellence,^44^ while Johnson and colleagues assert that effective community services should increase COC and reduce adverse outcomes.^45^ If service users had COC as defined from their perspective, they may feel they have high quality care; for instance, previous research has found that having service user‐defined COC in place predicts service user satisfaction.^16^ However, it is equally unlikely that service user‐defined COC contains all that people seek from high quality services. For example, while an in‐depth analysis of the focus group data found that professionals sometimes discriminated against service users,^46^ service user‐defined COC excludes the quality of staff relationships. Similarly, if professionals are able to deliver the components of care that comprise professional‐defined COC, they may feel that they are providing high quality services. However, it is also possible that service users do not have COC from their perspective but are seen to be in receipt of COC by providers e.g. where a crucial aspect of service user‐defined COC is absent. This underscores the importance of asking service users about their experiences through the lens of their own conceptualizations. It also supports Bachrach's distinction between continuous (i.e. non‐stop) care and COC,^32^ suggesting that quality is intrinsic to a definition of continuity.

### Towards a partnership paradigm and the use of PROMS

Heaton *et al*.^6^ argued that a recent UK health research programme on COC ‐ of which this study formed a component ‐ demonstrated a shift through Perspectivism towards a ‘Partnership Paradigm.’ However, further qualitative research is necessary to explore the co‐production of COC as it is enacted through relationships, underpinned by the conceptualizations of both service users and professionals. This could potentially include the use of PROMS (Patient Reported Outcome Measures), such as CONTINU‐UM. We have described the value of our method for generating measures of service users’ experiences elsewhere.^47^ Our experience is that grounding PROMS in extensive qualitative work with service users results in psychometrically robust measures that are important and relevant to other service users. We are particularly keen to highlight the valuable role of service user researchers in generating PROMS because this, to a certain extent, levels the power relations between researcher and participants. Levelling of power occurs because service user researchers are committed to transforming the role of those participating in research from traditional (passive) research subjects (research done to, on or for) to (active) participants (research done by or with),^48^ and more recently to co‐researchers involved in the interpretation of data and dissemination of findings^49^.’ It is National Institute for Health Research policy to encourage active Patient and Public Involvement (PPI) in research.^50^

### Strengths and limitations

In structure, the ECHO study comprised four distinct, complementary strands of work in which differing aims and objectives were achieved utilizing appropriate, reliable, valid methods, consistent with local study populations and conditions.^13^ Methodologies chosen to explore service user definitions of COC (facilitated focus group discussions; exploratory qualitative design) differed from those used to identify professional definitions (open question, self‐completion questionnaire, survey design). Although a synthesis of findings across ECHO strands was completed, the potential for crossover between some findings was not fully realized. Thus, our qualitative data emanated from the use of different methodologies to elicit service user and professional views and the fact that this may have influenced or explain some findings cannot be precluded. For instance, service users viewed day centres as important for COC, but these were not mentioned by health professionals. This difference may reflect different methodologies, or the inclusion of day centre attendees in the focus group sample; none of the health professionals worked in day centres although they would have been aware of the services offered outside the CMHT remit. To address this, future steps could include testing and amending the model utilizing the more cohesive Delphi study design.

Although unit and item questionnaire response rates were high in the professional survey, sampling the total population elicited a predominance of responses from nurses and social workers who constituted the dominant professional workforce groups; thus findings may not be representative of wider views of psychiatrists, psychologists and occupational therapists. With regard to bias due to gender and ethnicity, the majority of respondents were female (57%) and less than half (42%) were from minority ethnic backgrounds.

With regard to representativeness and potential bias in focus groups, both genders were represented (male participants predominated: 60%) and a range of ethnic backgrounds (White British ethnicity predominated: 75%), although fewer participants were from minority and mixed ethnic groups. A study information sheet disseminated through CMHTs invited interested service users to contact researchers directly if they wished to consider taking part, limiting bias. Criteria for inclusion ensured that those with complex needs and experiences of cross‐sectional and longitudinal COC were represented. The majority of participants were engaged with services, and it is acknowledged that those less well engaged may have different COC perspectives.

Although the primary aim of the service user research stream was to generate an outcome measure, the initial focus groups were entirely exploratory, focussing on participants’ experiences and definitions of COC. Repeat groups considered how COC could be measured, based on an exploratory thematic analysis of service users’ COC definitions and experiences, as expressed in the initial groups.

Important strengths of the ECHO study were its multidisciplinary perspective, encompassing strong inputs from service user researchers at all stages. INVOLVE, a government funded programme, supports patient and public involvement in the NHS, Public Health and social care research, bringing together insight, expertise and experience.^51^ Reflecting on the ECHO study experience, one service user researcher drew on a ‘double identity’ in understanding the experiences of participants, but was able to stand back and reflect on these using empirical research skills.

## Conclusion

This study has revealed important similarities and differences in the COC elements identified by service users and professionals in conceptualizing COC. Further research is necessary to explore these differences, prior to integration of service user and professional perspectives in a validated COC framework which could enable interventions to improve COC to be developed and evaluated. This would be consistent with the ‘Partnership Paradigm’^6^ in which the co‐production of COC as enacted in the concepts of both service users and professionals is supported. These future developments should be acknowledged and addressed by policy makers, service commissioners and researchers so that both professionals and service users’ actual needs drive and shape mental health services access, structure, organization and delivery. Yet it has recently been argued that, ‘an all‐encompassing definition that takes into account both the patients’ and professionals’ perspectives, makes COC something of a “bicephalous monster”’.^29^ Contrary to this, our findings suggest that to exclude either service users or professionals from a conceptualization of COC is to miss much of the picture.

## Conflicts of interest

No conflict of interest have been declared.
